# Introducing a recycling method for iron oxide from cold forming mild scale and its application in alkyd resin-based primer paint

**DOI:** 10.1038/s41598-026-47137-x

**Published:** 2026-07-21

**Authors:** Seyyed Majid Peighambari Sattari, Majid Kavanlouei, Seyyed Ahmadreza Khajehmohammadilar

**Affiliations:** 1 Department of Materials Engineering, Urmia University of Technology, Urmia, P.O.Box 57155-419, Urmia, Iran; 2 Department of Materials Science and Engineering, Faculty of Engineering, Urmia University, Urmia, Iran; 3 Research Centre for Advanced Materials, Faculty of Materials Engineering, Sahand University of Technology, P.O.Box 51335-1996, Tabriz, Iran

**Keywords:** Steel forming, Oxide scale, Pigment, Recycling, Corrosion resistance, Chemistry, Engineering, Environmental sciences, Materials science

## Abstract

Millions of tons of iron oxide-rich sludge waste are generated yearly by the steelmaking industry, posing significant environmental disposal challenges. This study aimed to develop a sustainable and economical technology by recycling this secondary material, specifically cold-forming oxide sludge, into a high-performance iron oxide pigment for use in corrosion-resistant coatings. The methodology involved a multi-stage purification process applied to the raw sludge: initial drying, separation of organic contaminants using a methanol solvent, magnetic separation to remove residual iron, and subsequent fine mechanical milling using a jet mill. The resulting powder was subjected to a crucial heat treatment at 400 ℃ to homogenize the microstructure. Finally, the refined iron oxide powder was mixed with an alkyd resin base and additives to successfully formulate a dark brown, liquid anti-rust and corrosion primer paint. The structural and chemical purity of the recovered pigment were characterized using XRD and XRF, while particle size and morphology were determined via DLS and SEM analysis, which indicated a particle size of approximately 50 to 100 nm. The performance of the final paint was rigorously validated against the ISIRI 4817 standard, employing tests such as salt spray (ASTM B117), pull-off adhesion (ASTM D4541), scratch resistance, humidity exposure, and resistance to petroleum solvents. The material characterization confirmed the successful recovery of the desired iron oxide phases (Fe_3_O_4_, Fe_2_O_3_, and Fe). The final primer paint demonstrated excellent barrier and mechanical properties, achieving a high adhesion rating (5B per ISO 2409), a satisfactory viscosity of 103.5 KU, and passing the 240-hour salt spray test with minimal defect rating. These results conclusively validate the feasibility of using waste steel sludge to produce a high-performance, anti-corrosion primer paint.

## Introduction

During the industrial production of different types of steel, tons of iron-rich mill scales are produced as waste materials^1^. Mill scale, which is the result of abrasion during metal forming in combination with industrial materials and greases, represents a substantial byproduct stream within the metallurgical sector^2^. The formation process typically involves high-temperature exposure during hot working or mechanical stress during cold forming, leading to the oxidation of the metal surface^3^. This surface layer, composed primarily of iron oxides (Fe_3_O_4_, FeO and Fe_2_O_3_) often incorporates remnants of cooling lubricants, rolling oils, and other processing aids used to manage friction and temperature during shaping operations^4^.

The waste from the steel industry, particularly mill scale, contains high levels of iron oxides and heavy metals that harm the environment when sent to landfills^5^. These contaminants can leach into soil and groundwater, threatening soil quality^6^. Additionally, the large amounts of mill scale take up valuable landfill space, increasing costs for steel producers and worsening the industry’s environmental impact^7^. The composition of this waste varies based on steel grade and processing methods, making management and recycling difficult^8^. To protect the environment and reduce disposal costs, it is vital to treat these wastes properly and advance new technologies for material production^9^. Adopting circular economy practices is essential for sustainability in heavy industry, highlighting the need for research in mill scale recycling^10^.

Some ways to reuse waste materials from the steel industry include using iron oxide pigments, new metal pieces, and chemical tankers^11,12^. Mill scale, due to its high iron content, can serve as a secondary source of iron, which can reduce the need for primary ore extraction^13,14^. An important use of iron oxide powder is as an additive in briquettes for blast furnace feeding^15^. When treated properly, mill scale can be turned into briquettes or pellets suitable for blast furnaces, as they contain very few harmful elements like sulfur and phosphorus, which can negatively affect metal quality^16^. Mill scales have the highest iron grade of steel industry wastes, often over 70% Fe content. They can also be used in electric arc furnaces (EAFs) to enhance slag foaming, which helps in energy efficiency and reduces electrode wear^17,18^. Additionally, they are used in secondary metallurgy to lower carbon, sulfur, and phosphorus levels during steel production^19^.

Research has focused on using mill scale to create valuable products due to its high iron oxide content^14,20^. The main component, magnetite (Fe_3_O_4_), is useful for magnetic separation and making pigments, especially in the ceramic color industry^21^. Iron oxide pigments are popular because they are low-cost, non-toxic, and stable^22^. Depending on the heating process, these can produce different colors, however, raw mill scale needs to be processed and purified, often requiring fine crushing and careful control over particle size and quality to achieve the desired pigments^23^. Other studies have looked at chemical processes to improve material quality^19,24^. For example, Mwebembezi et al.^25^, synthesized magnetic iron oxide nanoparticles from steel waste using an adsorption process for applications like water treatment. This process involves dissolving and reprecipitating the mill scale under controlled conditions. Liu et al.^26^ used Innovative methodology to produce ferrites samples. Although these methods yield high-quality materials, they can be complex and costly, raising challenges for industrial scaling and environmental management due to waste production.

The literature discusses how hot rolling processes produce scale that is mostly pure hematite (Fe_2_O_3_), as high temperatures promote its oxidized form^26^. This type of scale is well-understood and commonly used in sintering or as EAF charge. In contrast, there are fewer studies on mill scale from cold forming processes, like cold drawing or rolling, which generate thinner and oilier scales primarily made of magnetite (Fe_3_O_4_). These cold forming scales have higher organic content and finer particles, making them more like sludge than coarse powder.

This research focuses on using recycled pigment from oxide sludge to create corrosion-resistant alkyd-based primer paint. While alkyd coatings typically use high-purity iron oxide for color and protection, there is limited information on using waste-derived pigments. The study aims to ensure that this recycled material meets key performance standards, including opacity, tinting strength, and long-term corrosion inhibition. The research involves improving the quality of the pigment through techniques like mild thermal treatment or simple washing, avoiding costly chemical processes. The goal is to characterize the paint’s properties and corrosion performance, filling a gap in existing literature with a practical and economical approach.

Identifying a suitable method for recycling and reusing waste is important for economic, strategic, and environmental reasons. Economically, it helps reduce disposal costs and creates valuable materials. Strategically, it secures domestic sources of iron compounds, lessening reliance on mined ores. Environmentally, it reduces landfill pollution. This study focuses on optimizing the recycling of cold-forming oxide sludge to produce a high-purity pigment for performance alkyd primer paint (Table 1). The challenge is to transform complex, oil-contaminated sludge into a usable iron oxide pigment. The research aims to demonstrate feasibility, optimize thermal treatment, and validate the alkyd primer’s anticorrosive effectiveness.

**Table 1:** Comparison of previous pigment-from-mill-scale approaches and the present study.

Study/year	Feedstock source	Main processing route	Calcination/reaction temperature	Particle size	Intended application	Notable features	Limitation in previous work
Ammar et al., 2021	Mill scale from hot-rolling	Acid leaching + precipitation	700 °C	> 500 nm	Pigment powder	Simple acid recovery	High process temperature, coarse particles
Zhang et al., 2022	Mill scale	Hydrothermal oxidation + drying	600 °C	150–200 nm	Basic pigment	Moderate particle size control	Not evaluated for coating applications
Ramasamy et al., 2023	Mill scale + FeCl3 additive	Thermal oxidation	500 °C	200–250 nm	Pigment for ceramics	Uniform color shade	Complex reagent handling, limited coating tests
Al-Harahsheh et al., 2024	Steel oxide sludge + binder	Chemical conversion + filtration	650 °C	120–180 nm	Paint pigment	Improved purity	High energy input, solvent-based route
This work (present study)	Iron oxide sludge waste from cold-forming steel industry	Multi-stage recycling: organic separation → magnetic filtering → thermal treatment → pigment formulation	400 °C	50–100 nm (nano-scale)	Alkyd primer anti-corrosion coating	Low energy demand, simple route, eco-friendly, direct paint application	—

## Materials and methods

**Fig. 1:**
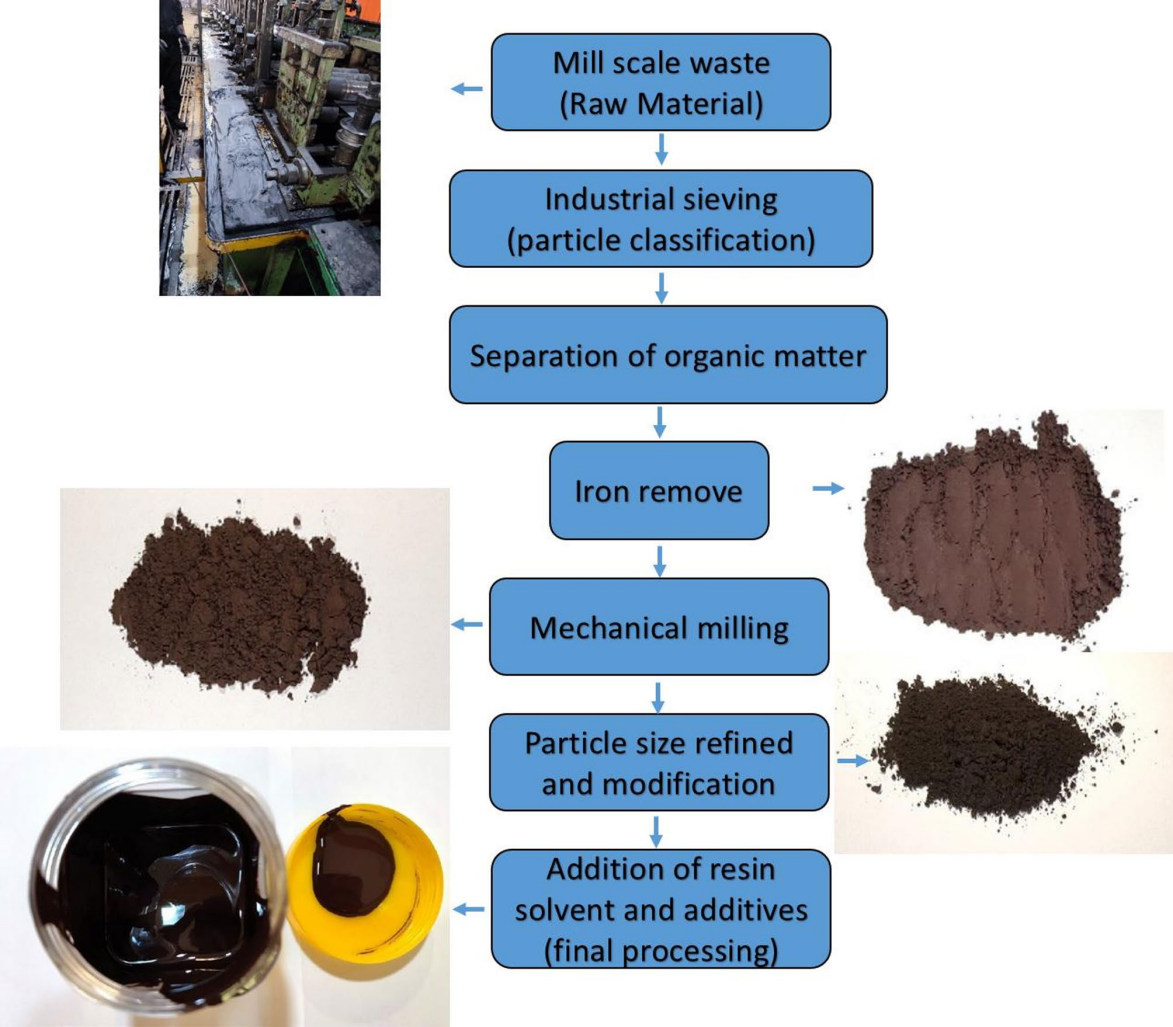
A roadmap of the approach used in this study.

**Fig. 2:**
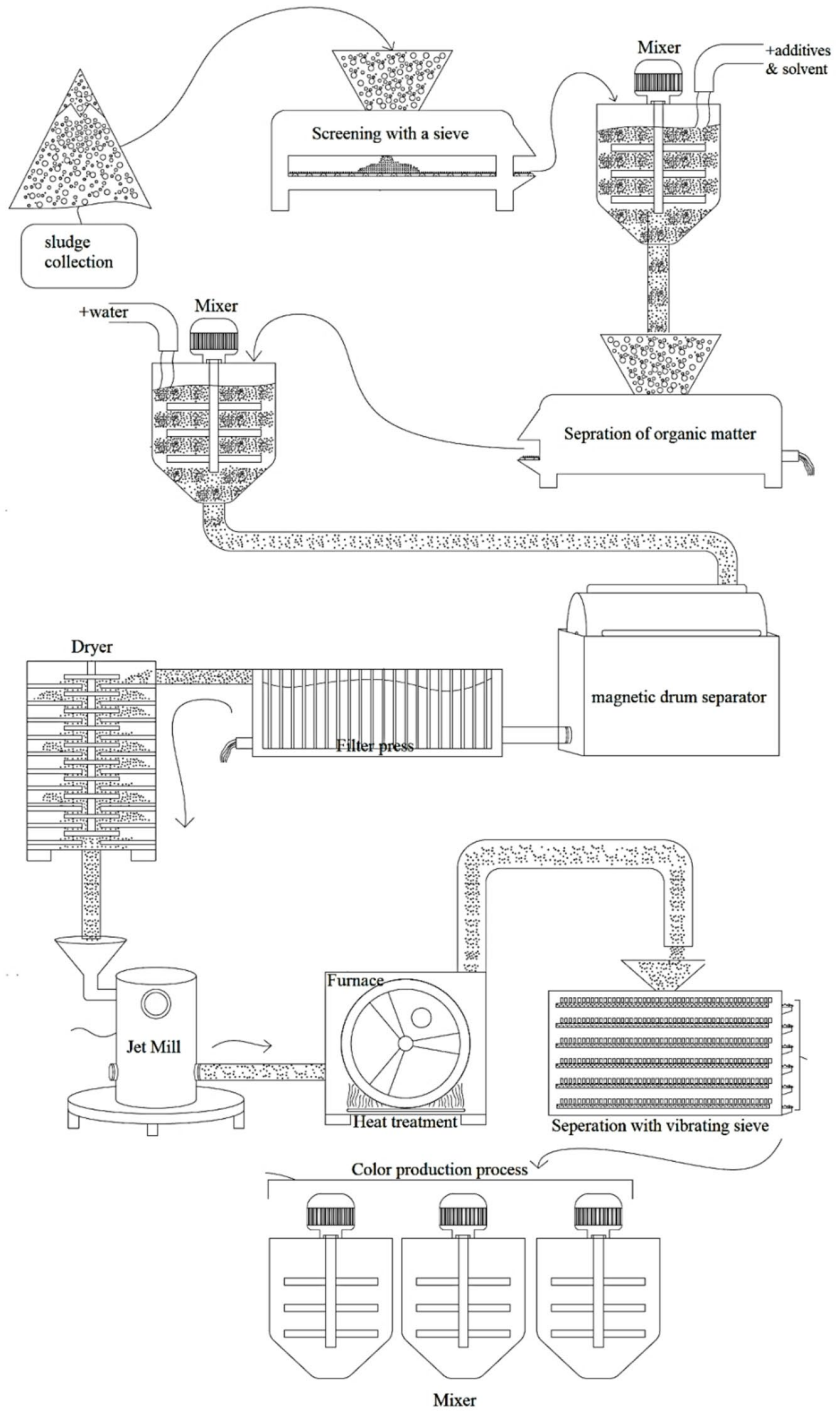
A schematic of the approach used in this study.

The production of the desired paint and pigment from mill scale involves several steps, as shown schematically in Fig. 1 (and further detailed in Fig. 2). This overall process includes: collection of mill scale, mechanical sieving, separation of organic matter, removal of non-magnetic/residual iron, fine mechanical milling, particle size modification, and finally, formulation with resin solvent and additives.

### Collection and initial purification

In the first step, the mill scale sludge resulting from the cold-forming process was collected. After collection, these materials were initially exposed to open air and sunlight for preliminary drying. Then, the dried materials were passed through a sieve to separate coarse materials. Specifically, industrial sieves with mesh sizes 3 and 4 were utilized in this stage to ensure the removal of larger particles.

### Organic matter separation

The next critical step was the separation of organic materials such as oil and grease. In this phase, the sludge was immersed in a pure methanol solvent and stirred for one hour to dissolve the organic contaminants. The samples were washed with methanol using a solid-to-liquid ratio of approximately 1:10 (g/mL) at room temperature (25 ± 2 °C) for three successive washing cycles.

The solvent mixture was then separated from the solid material using a filter press. Residual solvent (approximately 2 to 5%) was subsequently removed, and the material was dried. Methanol was used as a washing solvent due to its high volatility and ease of removal. In industrial applications, solvent recovery systems could be implemented to minimize solvent consumption and environmental impact.

### Magnetic separation and final dewatering

To increase the purity of the obtained powder, a magnetic separation method was employed using a drum magnet (specifically, a DDM Sep. SI-2201 model). The material was transferred to the drum, which effectively separates the magnetic particles (containing magnetite and iron) from the non-magnetic constituents. The resulting slurry was passed through special filter press cloths with a mesh size of 2500 to separate the water/solvent from the solid material, followed by drying in a dryer. Magnetic separation was carried out using a magnetic field strength of approximately 0.6–0.8 T, with a feed rate of about 2 kg h⁻¹. The process was repeated for two passes to improve separation efficiency.

### Milling and heat treatment

The dried materials then entered the jet mill (DS-LAB 10 kW) where grinding and pulverizing operations were carried out for about 45 to 50 min to achieve the desired initial grain size. Jet milling was performed at an operating pressure of approximately 6 bar, with a classifier speed in the range of 3000 rpm and a feed rate of about 1 kg h⁻¹. Subsequently, the powders were subjected to a crucial heat treatment and oxidation process in a furnace at 400 ℃ for a period of 30 to 40 min under a pressure of 1 to 1.5 bar. This step was paramount for homogenizing the crystallographic microstructure and eliminating residual iron. The heat treatment was performed in air with a heating rate of 5 °C min⁻¹, followed by natural cooling inside the furnace. A slightly elevated and controlled pressure (1–1.5 bar) was maintained to ensure stable airflow conditions and safe furnace operation, rather than to induce any pressure-driven thermochemical effects. Also, The selected heat treatment temperature (400 °C) is sufficient to promote oxidation of iron phases present in mill scale while preventing excessive grain growth or sintering of particles.

### Final particle size control and formulation

Following heat treatment, minor granulation was performed, and the powders were separated again using a laboratory screening machine to ensure the particle size was suitable for pigment application. After achieving crystallographic microstructural and chemical homogeneity, the iron oxide powder was mixed with additives (according to Table 2) in various stages to produce a liquid anti-corrosion and rust paint. Finally, the appropriate formula was obtained, resulting in a dark brown liquid anti-rust and corrosion paint. A schematic of this complete approach is shown in Fig. 2.

**Table 2:** Color combination in this study.

Material name	Content (in 1000 cc)
Long Oil 70%, grade 1, AZAR ZARRIN Co. (IRAN)	280
Soybean lecithin, a well-known surface active agent	10
Anti-fouling gel 340 10%	100
iron oxide	150
Talc	150
Solvent 402 - Petro Refinery Gahar Lorestan Co.	90
Calcium carbonate	210
Calcium Chloride Desiccant 10%	5
Lead improves flexibility and durability of the film 32%	5

### Final characterizations

The produced samples were then analyzed through X-ray diffraction with the Bruker Advance D8 advanced instrument (Germany), by applying Cu Kα radiation (λ = 1.5406 Å) at 50 KV and 250 mA in the two theta ranges of 20°–80°. The X’pert high score plus software was then used for phase identification and microstructural study through the Rietveld refinement method. Dynamic light scattering (DLS) analysis was performed to measure the submicron particle size of samples before and after ultrafine grinding by the HORIBA Scientific, SZ-100 instrument. The particle size distribution obtained from DLS measurements was reported in terms of D10, D50, and D90 values. The microstructure of powders was studied by SEM (Leica Cambridge, Stereoscan S360), FESEM (MIRA3TESCAN-XMU) and EDAX were used to determine the surface morphology and chemical composition of the powders. Particle size histograms were constructed from SEM images by analyzing more than 100 individual particles using ImageJ software. Chemical analysis was performed using XRF tests according to the standards BS EN 13925-1: 2003^27^ and ASTM E1621-13^28^, respectively. To investigate the corrosion resistance and usability of this paint, a salt spray test was performed on the paint made from the pigments according to the standard ASTM B117 – Standard Practice for Operating Salt Spray (Fog) Apparatus^29^. The pull-off test was also performed according to the standard ASTM D4541 – Standard Test Method for Pull-Off Strength of Coatings^30^. In addition, the particle size test was performed during the manufacturing process according to the standard ASTM D1210^31^. Finally, scratch resistance, cold water, humidity, petroleum solvents, and sealing resistance tests, as well as adhesion and bending tests, brush and spray application capabilities, and other tests required to investigate the application of this paint as an anti-rust and corrosion paint, were performed according to the standard ISIRI 4817 at 25 degrees Celsius^32^. The quality and performance characteristics of the final alkyd-based primer were evaluated following standard testing protocols. The viscosity of the undiluted paint was measured using a Krebs-Stormer viscometer. The acceptable industrial range targeted was 95–115 Krebs Units (KU).m. Adhesion to the prepared steel substrate was assessed using a Cross-Cut Test as per ISO 2409 – Paints and Varnishes — Cross‑Cut Test. Bending resistance was determined by assessing the film’s integrity (cracking/loss of adhesion) after being mechanically bent around a specified mandrel diameter. Samples were subjected to immersion in cold water and high humidity conditions for 24 h to evaluate moisture barrier performance and adhesion retention. Resistance to skinning was verified by sealing the paint sample (75% fill) in a container and storing it at 25 ℃ for 48 h. The resistance to common petroleum solvents was tested by exposing the cured film to a cotton swab saturated with AW402 solvent for a contact time of 2 h.

## Results and discussion

**Fig. 3:**
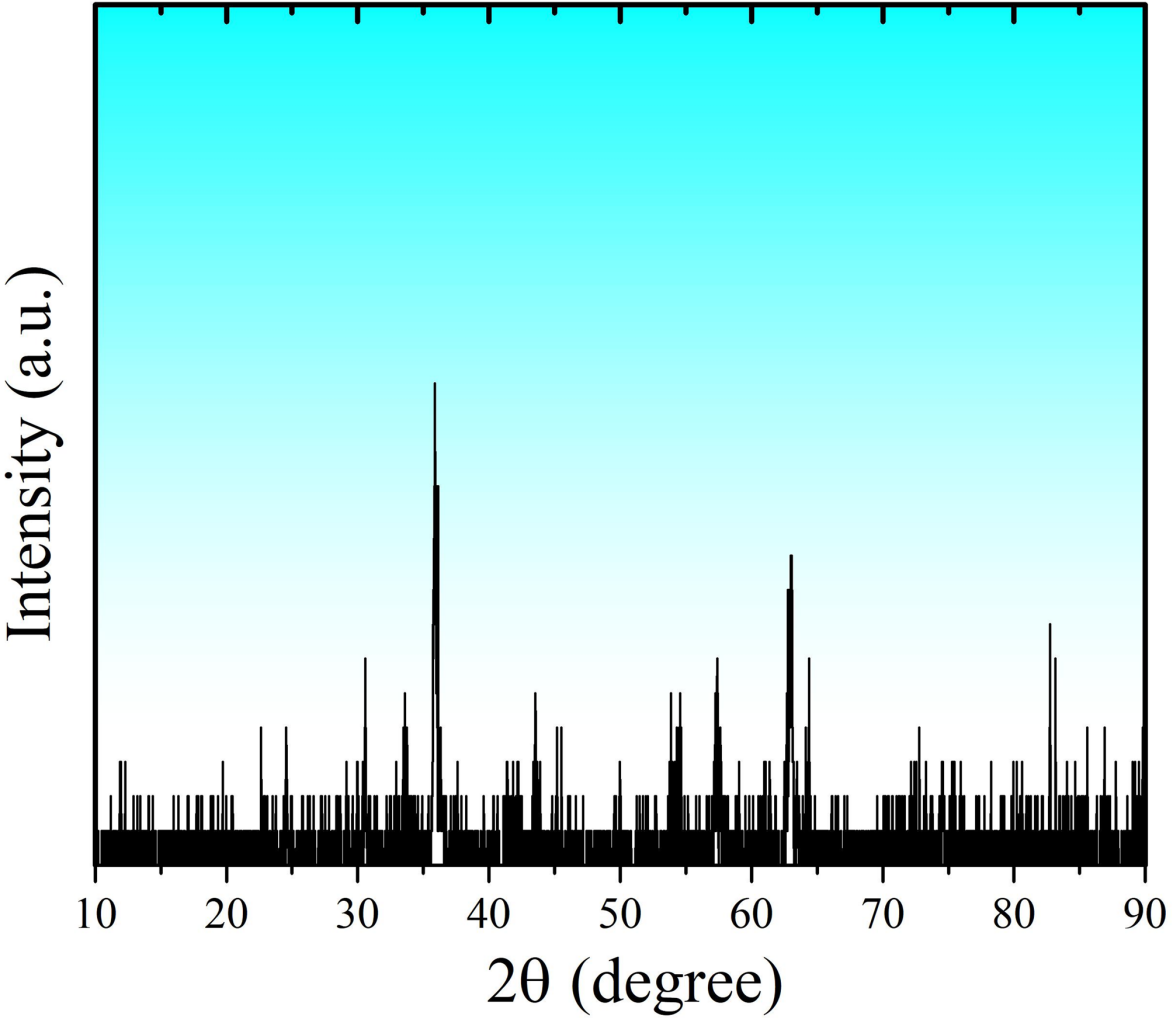
XRD diffraction pattern of product before removal of organic pollutants.

**Fig. 4:**
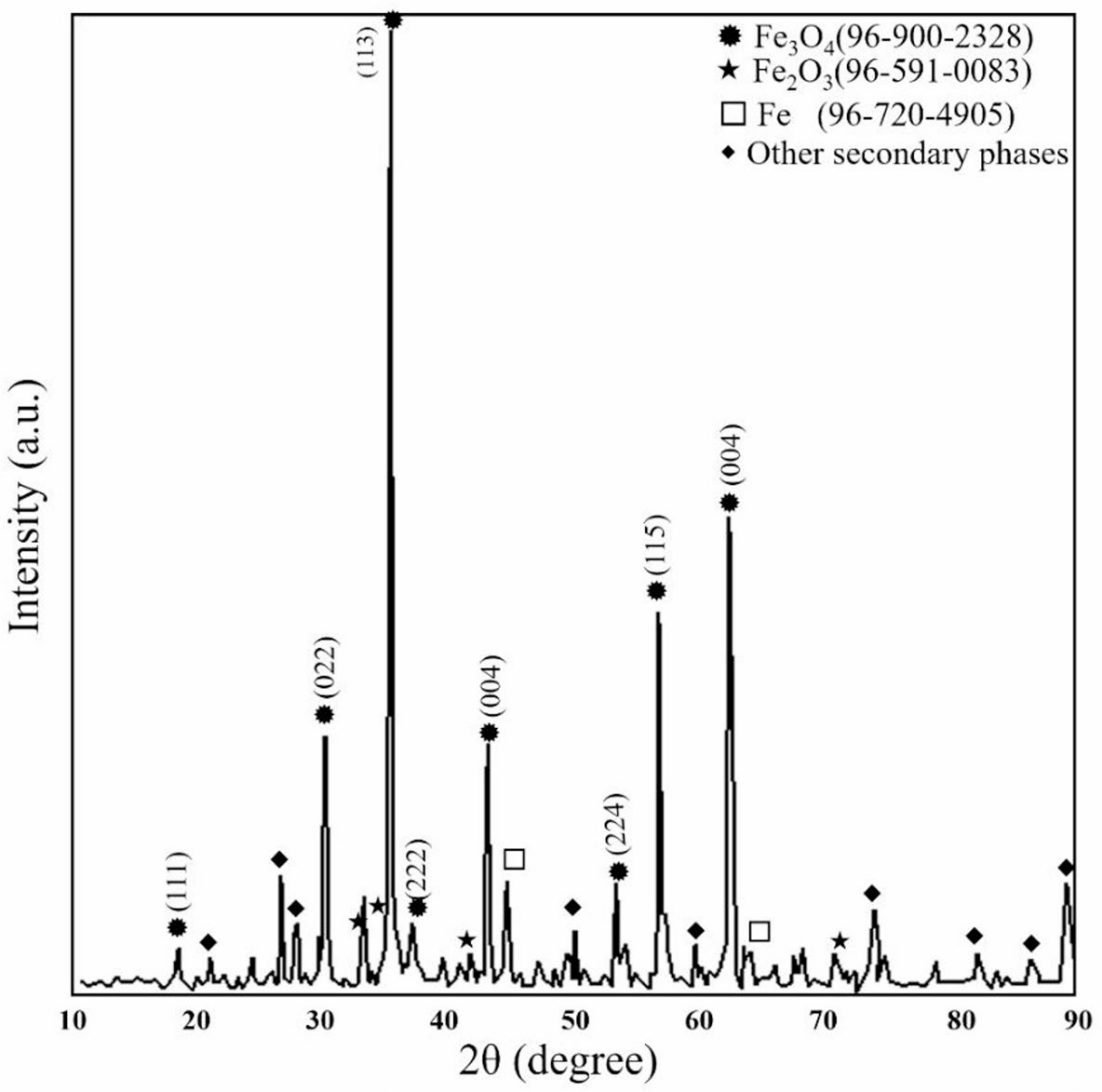
XRD diffraction pattern of product after removal of organic pollutants.

**Fig. 5:**
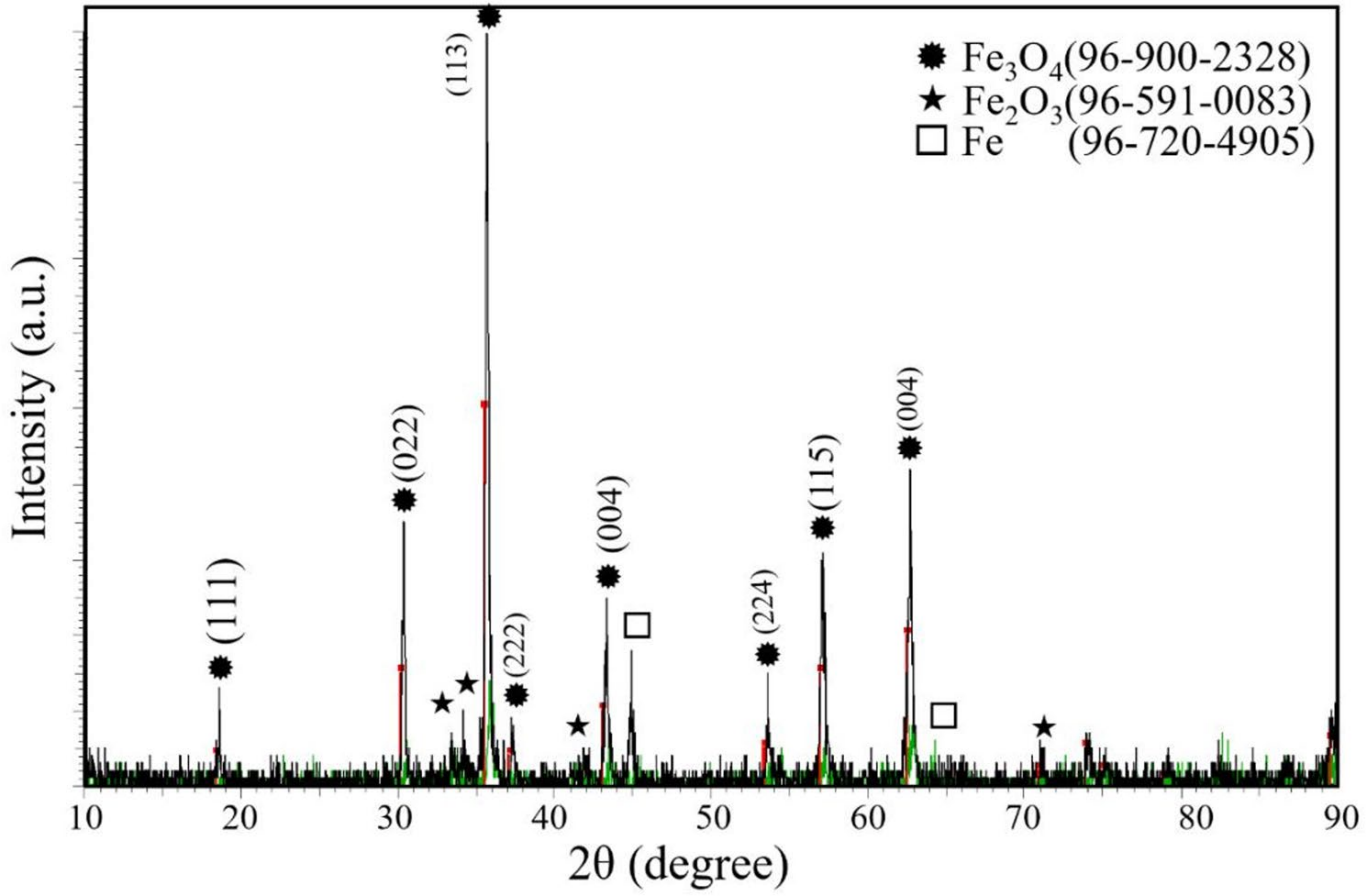
XRD diffraction pattern of product after removal of organic pollutants and drum magnet process.

Collection of waste and primary screening to separate impurities was carried out using conventional methods. Due to the long period of processes, the capacity of related installations can be reduced and energy consumption and costs may be increased. Therefore, stirred and milling times were preferred to be less than one hour during the production in this study, thus overcoming the hindering impacts of the above-mentioned problems. The diffraction images of raw powders in this step are also shown in Figs. 3, 4 and 5. The reflection peaks of Fe (JCPDS No. 96-720-4905, Cubic, Space group: Im-3 m) and Fe_2_O_3_ (JCPDS No. 96-591-0083, Hexagonal, Space group: R-3c) and Fe_3_O_4_ (JCPDS No. 96-900-2328, Spinel) were visible even after purification and after cleaning. No additional crystalline phases other than Fe₃O₄, minor Fe₂O₃, and metallic Fe were detected. The residual Fe metallic phase is attributed to incomplete oxidation of Fe-rich regions within the mill scale particles. In dense particles, iron can remain partially encapsulated by oxide layers, limiting oxygen diffusion during heat treatment at 400 °C. The results of XRD analysis in two stages, after purification and cleaning, are respectively Magnetite, Hematite, Iron and Magnetite, Iron. As can be observed, the XRD pattern prior to treatment exhibits additional diffraction features and an elevated background intensity, which can be attributed to the presence of residual organic species on the surface and within the structure of the material. These organic components partially mask the characteristic diffraction peaks of the inorganic phase. After the removal of organic pollutants, the XRD patterns (Figs. 4 and 5) display sharper and better-defined diffraction peaks with reduced background noise, indicating an improvement in crystallinity and phase purity. No new crystalline phases were detected after treatment, confirming that the removal process did not alter the intrinsic crystal structure of the material. The clearer visibility and increased intensity of the characteristic peaks after treatment demonstrate the effective elimination of organic residues, thereby validating the efficiency of the organic pollutant removal process.

**Fig. 6:**
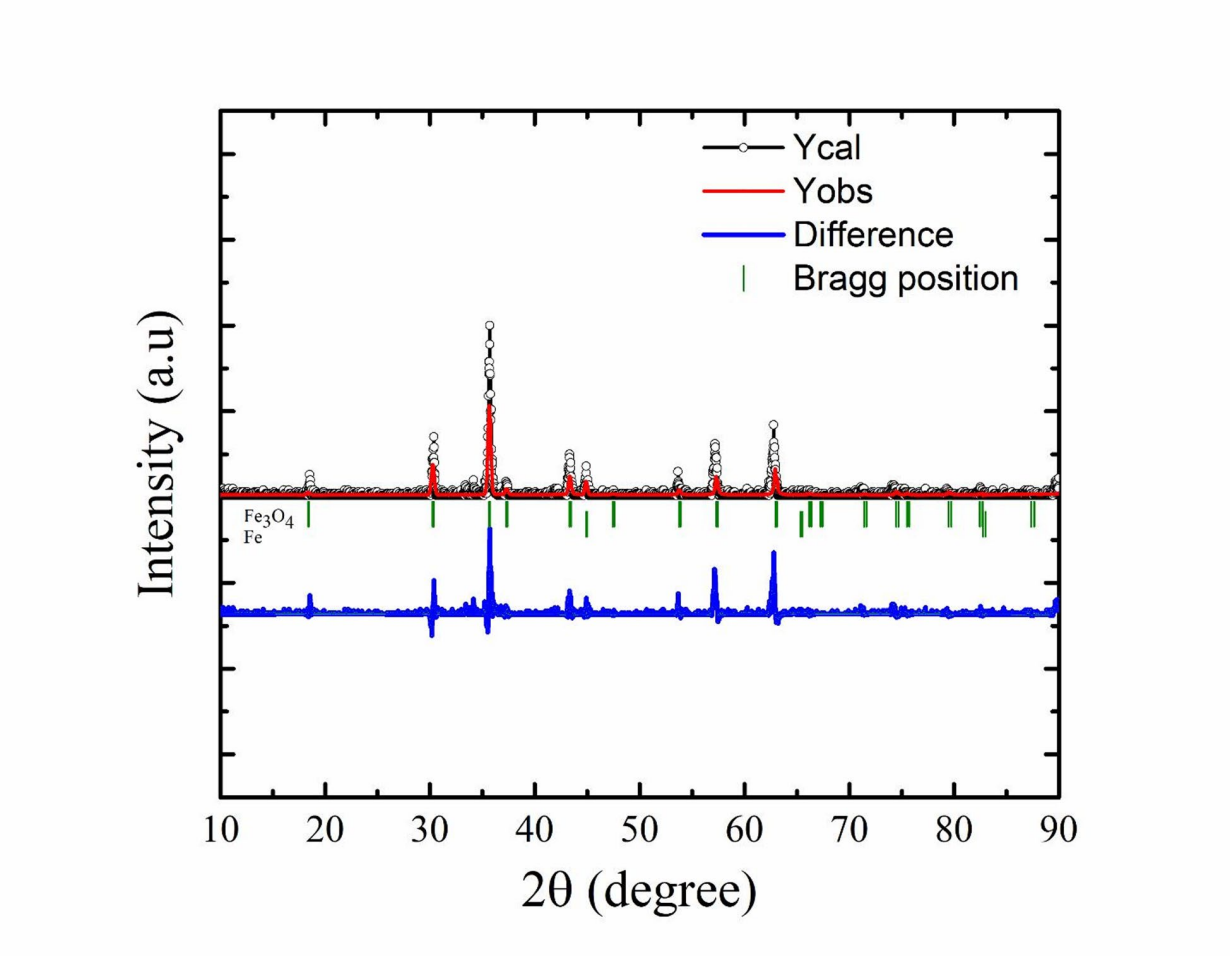
Experimental X-ray powder diffraction pattern (dotted curve) of product after removal of organic pollutants and drum magnet process compared to the Rietveld–refined profile) (continuous line).

Also, Changes in peak intensity with different phases indicate the percentage of various phases. Therefore, Rietveld analysis was used for a more detailed study of the microstructural features. The related XRD patterns of all powders which had been milled were analyzed by the Rietveld method. It can be seen that the observed and calculated patterns are in good agreement. The related values of the R-factors are less than 20% between the observed pattern and the refined pattern, which confirms the refinement quality. Also, lattice parameter changes were also obtained using Rietveld analysis. The results of phase analysis in Rietveld method (for XRD pattern Fig. 5) calculated that the amount of Fe_3_O_4_, Fe_2_O_3_ and Fe phase in the powder mixture 94.94, 0.9 and 4.14 wt%, respectively (as shown in Fig. 6). Considering its relatively low fraction (~ 4 wt%), the presence of metallic Fe is not expected to significantly affect the overall pigment performance. However, localized oxidation of metallic Fe in service environments may occur and could potentially influence long-term coating behavior.

**Fig. 7:**
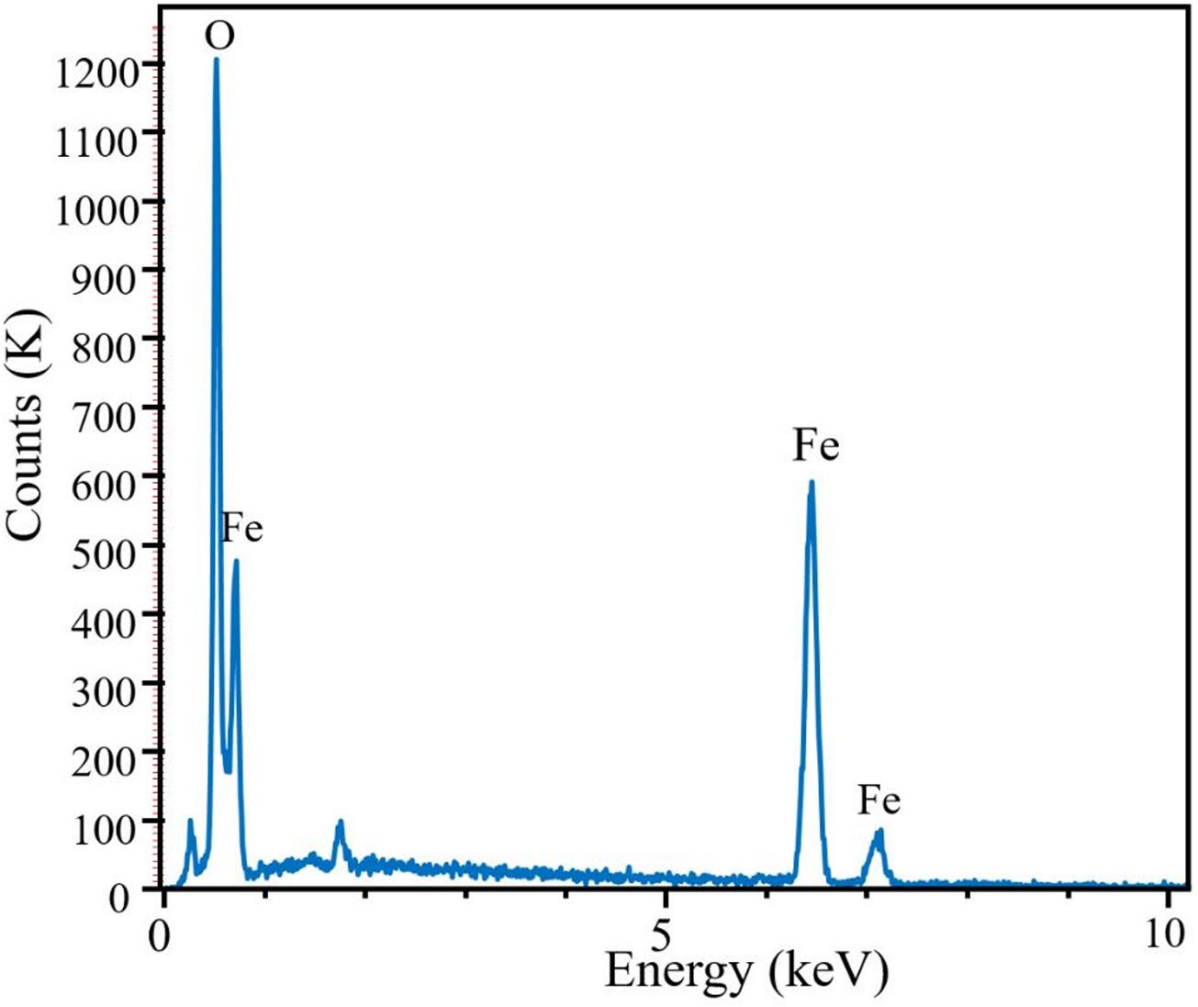
EDAX results for final nanopowders.

Furthermore, an EDAX analysis of the produced samples yielded invaluable insights into the elemental composition of the powder surface. The EDAX analysis, depicted in Fig. 7, revealed that discernible Fe impurities were evident in the produced powder; furthermore, alongside the primary material peaks encompassing Fe, only oxygen presence was detected. A significant aspect was the presence of oxygen contamination in the raw powder (as observed via Quantitative EDAX analysis Table 3), which can be due to the presence of surface oxides on the particles. The results of the EDAX analysis seamlessly aligned with the conclusions drawn from the Rietveld analysis. The EDAX results also confirm the previous results of the XRD diffraction pattern in the absence of impurities and secondary phases.

**Table 3:** Quantitative EDAX Results for final nanopowders.

Elements	Intensity	Weight% (Wt%)
C	60.8	11.78
O	347.7	35.20
Na	57.3	10.42
Cl	76.4	4.24
Fe	615.2	38.35
		100.00

The result of XRF test after the purification step is presented in Table 4. As can be seen in Tables 1 and 2, the purity of the particles after the purification step is about 98%. However, due to the presence of free iron in the resulting composition, the quality of the produced powder is not suitable for use as a pigment. Free iron reduces the anti-corrosion properties of the paint. Therefore, to improve the quality of the pigment, free iron is removed from the pigment through metallurgical processes. The presence of oxides listed in Table 4 is also controlled and does not cause any undesirable properties in the paint.

**Table 4:** Result of XRF for the before drum magnet process, after drum magnet process and final nanopowders.

Before drum magnet process		After drum magnet process		Final nanopowders	
Oxides	W%	Oxides	W%	Oxides	W%
Fe_2_O_3_	93	Fe_2_O_3_+FeO	96.43	Fe_2_O_3_+FeO	98.05
SiO_2_	3.94	SiO_2_	1.8	SiO_2_	0.85
Al_2_O_3_	0.64	Al_2_O_3_	0.6	Al_2_O_3_	0.3
CaO	0.28	CaO	0.4	CaO	0.3
MnO_2_	0.41	MnO_2_	0.43	MnO_2_	0.46
SO_3_	0.2	SO_3_	0.05	SO_3_	0.03
K_2_O	0.05	K_2_O	0.01	K_2_O	0.01
L.O.I	0	L.O.I	0	L.O.I	0

**Fig. 8:**
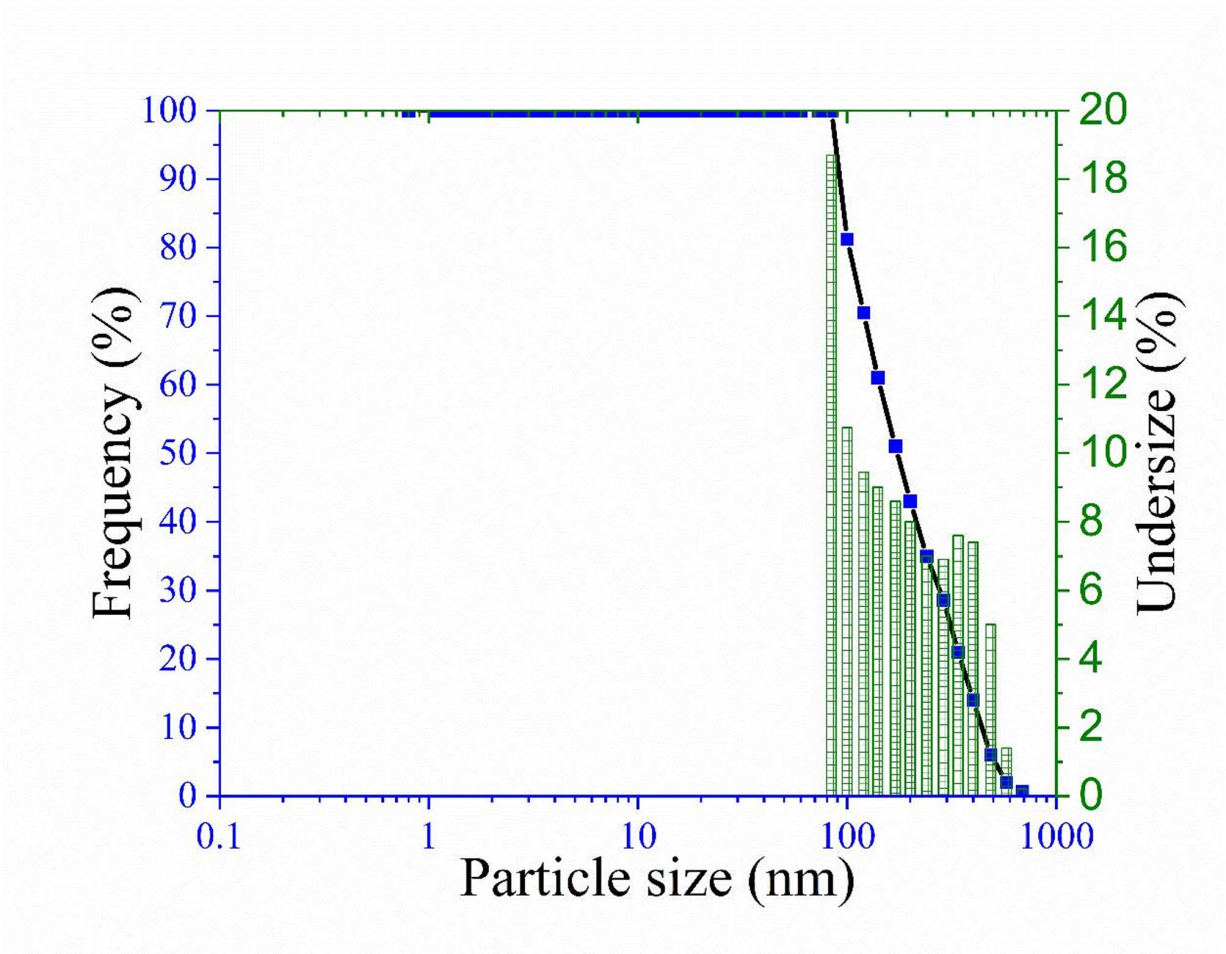
The results of the particle size distribution of the final nanopowders.

The results of the DLS test are shown in Fig. 8. As can be seen from this figure, the size of the particles is below 1000 nm. The particle size distribution obtained from DLS measurements was characterized by D10, D50, and D90 values of 100 nm, 170 nm, and 450 nm, respectively. In the following, since the pigments have much smaller sizes than the obtained powder, the particle size distribution was investigated to reduce the size to the nanometer scale. To reduce the size to the nanometer scale, a combination of multi-stage ball mill and jet mill was used, and then the particle size was analyzed using the DLS method.

**Fig. 9:**
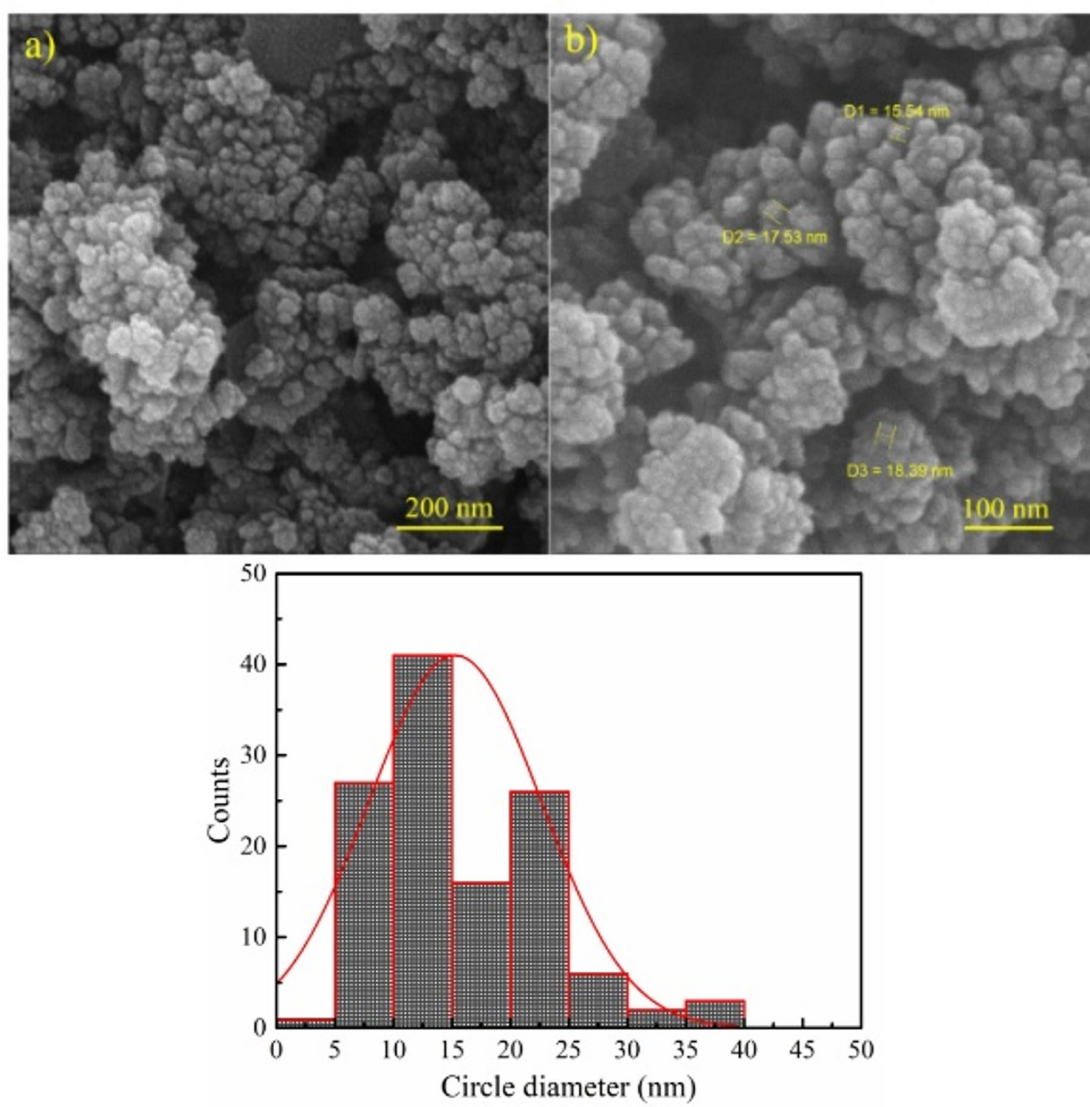
(**a**) SEM micrograph of the synthesized sample after the removal of organic pollutants, showing the overall surface morphology. (**b**) Magnified view of the selected region marked in (**a**), illustrating the nanoscale features of the material. (**c**) Particle size histograms were constructed from SEM images by analyzing more than 100 individual particles using ImageJ software.

Figure 9 shows the SEM images of produced sample. The results show a relatively uniform particle size distribution of 100 to 1000 nanometers. Due to the limitations of the DLS method in measuring nanoparticles, SEM was used to investigate the morphology and size of the particles, which showed that the particles have a size of about 50 to 100 nanometers and are spherical in shape with good uniformity. The particle size difference of DLS with the SEM images can be due to the difference in the measurement method. In DLS, the refractive index of the particles is used and the particles are dispersed in a solution. Figure 9a presents the general surface morphology of the sample after the removal of organic pollutants. To provide a clearer insight into the nanoscale characteristics, a magnified image of the selected area is shown in Fig. 9b. The enlarged view reveals a relatively uniform distribution of nanoparticles. The particle size was quantitatively evaluated using ImageJ software, confirming that the observed features are within the nanoscale range. This magnified analysis supports the morphological observations and provides a more accurate assessment of particle size and distribution. Particle size histograms were constructed from SEM images by analyzing more than 100 individual particles using ImageJ software (Fig. 9c). The larger particle sizes observed in DLS measurements compared to SEM observations are attributed to particle aggregation in suspension, which is a well-known limitation of light-scattering-based techniques for fine powder. By correlating DLS statistical data with direct SEM observations, a consistent particle size range was established, confirming the effectiveness of the milling process.

**Fig. 10:**
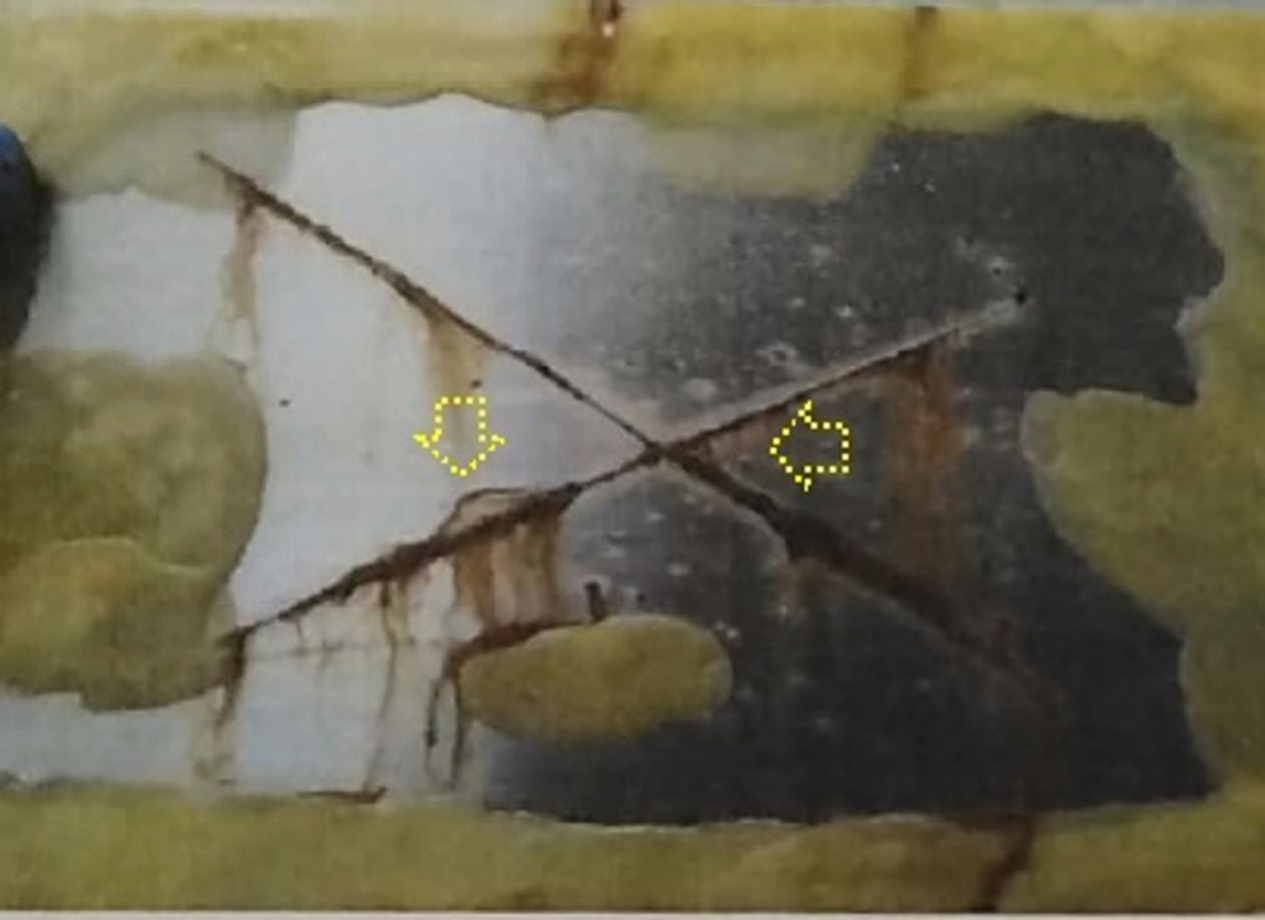
Results of the salt spray test after 240 h exposure, showing the surface condition of the coated panels following continuous exposure to a neutral salt fog. (Arrow: coating (painted) area/coating boundary, Scribe line: intentionally scratched (scribe) region, ×: marked evaluation point/selected inspection location)

The performance of the anti-rust primer under aggressive corrosive conditions is critically demonstrated by the Salt Spray Test results after 240 h, visualized in Fig. 10. Following exposure to a constant fog of neutral salt solution, the protective capacity of the paint film was rigorously assessed using two primary standards. The first evaluation, according to ASTM D714, focused on blistering. Critically, no blistering was observed on any of the tested panels, indicating that the recycled pigment/alkyd matrix successfully prevented the penetration of corrosive species to the substrate interface. Subsequent examination for delamination and coating failure, adhering to ASTM D1654, resulted in a high rating score of 6 (on a scale of 0 to 10, where 10 is flawless). This rating confirms that the coating integrity remained largely intact, with minimal localized defects, well below the threshold of significant failure (which is 75% defect at a score of 0). Furthermore, complementary mechanical tests showed a pull-off adhesion strength of 0.80 MPa, confirming that the electrochemical protection did not compromise the paint’s physical bond to the metal. Taken together with the successful particle size reduction during processing (from 60 μm after one hour to 40 μm after two hours, meeting the desirable fineness), these results validate the formula’s superior ability to both resist corrosion initiation and maintain strong barrier properties over extended exposure periods.

Finally, the quality test of the paint was also performed, the results of which are fully presented in Table 5. The obtained anti-rust paint obtained a result of 5B in the adhesion test, which indicates that the amount of paint separated from the metal surface is 0%. In the resistance test to closing the lid, the prepared paint sample was poured into 75% of the container, then the container was tightly closed and placed at 25 degrees Celsius for 48 h. After the mentioned time, no skinning was observed in the anti-rust primer. The viscosity test is performed using a Krebs-Stormer viscometer to determine the viscosity of the undiluted paint. The acceptable range for this test is 95–115 Krebs units. If the viscosity is higher or lower than this range, the paint will not flow properly on the desired surface. In this test, the result was 103.5 Krebs units, which is a desirable result for a paint. The paint also showed good bending resistance in the bending test, which is an important characteristic in relation to the surface quality of the paint, especially when the substrate is expanding or contracting and the paint should not crack. This paint did not blister or lose adhesion after being kept in cold water and humidity for 24 h, which is one of the characteristics of anti-rust and corrosion resistance of the paint. The paint showed good resistance to petroleum solvents. In such a way that after two hours of exposure to cotton soaked in AW402 solvent, no traces of primer pigment were observed on the cotton soaked in the solvent, and no visible defects such as softening and blistering were observed. It has been demonstrated that iron-rich mill scale can be processed into high-quality oxide pigments with particle sizes in the nanoscale range (e.g., 0.18–0.3 μm) and controlled morphology^33^. In line with these findings, the present work reports on the synthesis and characterization of pigments derived from similar waste streams, emphasizing particle size control and surface area properties.

The Performance and Quality Testing of the Final Primer assurance tests detailed in Table 5 (AS 3730.21–2006 standard), confirmed the high performance profile of the primer formulated with the recycled mill scale pigment. The adhesion test yielded a result of 5B according to the cross-cut methodology (ISO 2409). This result signifies 100% retention of the paint film on the substrate post-taping, indicating superior mechanical bonding. Furthermore, the bending test confirmed excellent film flexibility, preventing cracking under substrate deformation, which is critical for long-term protection. Rheological analysis showed the paint viscosity to be 103.5 KU. This value lies centrally within the acceptable range of 95–115 KU, ensuring optimal flow, leveling, and application characteristics without risk of sagging or poor coverage. Stability tests were also successful. The paint showed no evidence of skinning after 48 h of sealed storage, indicating proper stabilization. Crucially, the barrier properties were validated by the absence of blistering or adhesion loss after 24 h of exposure to cold water and humidity, confirming effective anti-corrosion capabilities. Finally, the primer demonstrated robust resistance to AW402 petroleum solvent, with no pigment leaching or film softening observed after two hours of exposure, validating the integrity of the cured alkyd matrix.

While this investigation successfully demonstrated the feasibility of recycling iron oxide-rich sludge into a functional anti-rust primer, certain limitations warrant consideration. The salt spray testing, a standard indicator for corrosion resistance, was conducted for 240 h according to ASTM B117. While this duration provides a solid baseline performance metric, it does not encompass the long-term durability required for demanding applications or exposure to varied climatic conditions. Furthermore, the current study was primarily conducted at a laboratory scale, focusing on process optimization and initial formulation validation. Scaling up the recovery and pigment production processes to industrial levels may present unique challenges related to material handling, energy consumption, and consistent quality control that require further investigation. Additionally, while the formulated primer exhibited good adhesion and initial protective properties, a more comprehensive evaluation including cyclic corrosion tests, weathering studies under different environmental parameters (e.g., UV radiation, humidity fluctuations), and investigation into alternative binder systems could further enhance the understanding of its long-term protective efficacy. Future research should aim to address these aspects to fully delineate the potential of this recycled pigment in high-performance coating systems. A detailed energy consumption analysis was beyond the scope of this study and will be addressed in future scale-up investigations.

**Table 5:** Quality test of the paint.

Test name	Acceptable range	Result
Compliance with anti-rust conditions in the can	Yes	Yes
Dilatability with solvent	Acceptable	Acceptable
Workability with brush	Acceptable	Acceptable
Workability with spray gun	Acceptable	Acceptable
Resistance to closing film	Resistant	Resistant
Density (g/cm3)	Maximum 1.6	1.53
Maximum particle size (microns)	40	30
Flexibility	No cracking	No cracking
Adhesion (4B minimum)	5B	4B
Viscosity at 25 °C	95–115	103.5
Surface drying time	Max 45 min	15 min
Full drying time	Max 3 h	2 h
Scratch resistance	Minimum 1500 g	Resistant
Cold water resistance	Minimum 24 h	Resistant
Humidity resistance	Minimum 24 h	Resistant
Resistance to petroleum solvent	Minimum 2 h	Resistant
Minimum weight% of non-volatile matter	65%	77.03%
Minimum weight% of dry resin	12%	18.49%

## Conclusion

This research demonstrates a viable and environmentally responsible pathway for utilizing iron oxide sludge waste generated from cold-forming steel industries. The multi-stage recycling process, involving organic separation, magnetic filtering, and thermal treatment, transformed the complex sludge into a high-purity iron oxide pigment with particle sizes controlled within the range of 50 to 100 nanometers, as confirmed by SEM and DLS analyses. The approach further enabled the formulation of a dark brown, alkyd resin-based primer paint. Performance evaluation according to ISIRI 4817 and complementary international standards (ASTM and ISO) showed satisfactory results. The paint exhibited good barrier properties, showing no blistering after 240 h of salt spray exposure and maintaining strong adhesion (5B rating) and delamination resistance (score of 6). The film also demonstrated good flexibility (passing the bending test), appropriate rheological properties (103.5 KU viscosity), and resistance to cold water, humidity, and petroleum solvents (AW402). Overall, the recovered iron oxide pigment shows potential for use in anti-rust and corrosion-resistant primer coatings. These findings highlight the potential of recycling steel industry sludge as a functional pigment source, contributing to circular economy strategies by converting industrial waste into a value-added material.

## Data Availability

Data will be made available on request by sending an email to corresponding author.
